# Molecular association of *Candida albicans* and vulvovaginal candidiasis: focusing on a solution

**DOI:** 10.3389/fcimb.2023.1245808

**Published:** 2023-10-13

**Authors:** Helma David, Adline Princy Solomon

**Affiliations:** Quorum Sensing Laboratory, Centre for Research in Infectious Diseases (CRID), School of Chemical and Biotechnology, SASTRA Deemed to be University, Thanjavur, India

**Keywords:** *C. albicans*, vaginal candidiasis, predisposition factors, morphogenesis, multidrug resistance, possible drug targets

## Abstract

*Candida albicans*-mediated vulvovaginal candidiasis (VVC) is a significant challenge in clinical settings, owing to the inefficacy of current antifungals in modulating virulence, development of resistance, and poor penetration into the biofilm matrix. Various predisposition factors are molecular drivers that lead to the dysbiosis of normal microflora of the vagina, upregulation of central metabolic pathways, morphogenesis, hyphal extension, adhesion, invasion, and biofilm formation leading to chronic infection and recurrence. Hence, it is crucial to understand the molecular mechanism behind the virulence pathways driven by those drivers to decode the drug targets. Finding innovative solutions targeting fungal virulence/biofilm may potentiate the antifungals at low concentrations without affecting the recurrence of resistance. With this background, the present review details the critical molecular drivers and associated network of virulence pathways, possible drug targets, target-specific inhibitors, and probable mode of drug delivery to cross the preclinical phase by appropriate *in vivo* models.

## 1 Introduction: VVC

Vulvovaginal candidiasis (VVC) is an inflammatory mucosal infection in the lower reproductive tracts of women. Among women of different ages, more reported cases of VVC are in the reproductive age. Also, 75% of women experience at least one episode of VVC in their lifetime, and 8% experience a recurrence of infection, at least four episodes per year. Global statistics show that 138 million women worldwide experience recurrent VVC each year, with an annual prevalence of 3,871/100,000. According to base case estimates considering the increase in female population worldwide, the expected number of individuals affected with recurrent VVC is predicted to rise to over 158 million by 2030. As a result, 20,240,664 more VVC-affected cases are added to the predicted increase in female population from 3.34 to 4.181 billion people (Żyrek et al., 2021).

The global statistics of VVC are hiked by *Candida*, a predominant fungal colonizer of the vaginal lumen (Ahangari et al., 2019). *Candida albicans* and several other related species, *Candida glabrata*, *Candida tropicalis*, *Candida parapsilosis*, and *Candida krusei* cause VVC (Guinea, 2014). Interestingly, *C. albicans*, the primary causative agent of VVC, is categorized as a critical fungal priority pathogen (CFPP) by the WHO (World Health Organization) (Fisher and Denning, 2023). *C. albicans* is also a benevolent partner of the microbiome of healthy individuals and is capable of switching from a commensal to a pathogenic state under adverse conditions driven by various predisposition factors (Mukaremera et al., 2017). Several predisposition factors are associated with VVC, which include diabetes mellitus (DM), elevated endogenous estrogens (from pregnancy or obesity), immunosuppression (i.e., transplant patients, antimetabolite medications or chemotherapy, or HIV infection), broad-spectrum antibiotic use, and other environmental factors (Gonçalves et al., 2016). Recent studies have shown that patients with DM are more prone to candidiasis, as glucose plays a significant role in the colonization and proliferation of these pathogens in the host (Gürsoy et al., 2018). Host-influenced (predisposition) factors are critical in driving *C. albicans* morphogenetic lifestyle changes from yeast to hyphal and enable it to cross its boundary from commensal to pathogenic form and *vice versa* (Mukaremera et al., 2017). *C. albicans*, in its lifestyle transition, express various virulence traits that increase its survival fitness in the host and lead to the rapid evolution of resistance to antifungals (Slutsky et al., 1985; Sudbery et al., 2004). The survival fitness of *C. albicans* enhances them to effectively colonize the host and leads to the spread of infection in an asymptomatic/symptomatic mode causing severe mucosal inflammation in affected individuals (Farhan et al., 2019). Mucosal inflammation is a symptomatic response in patients who may exhibit vaginal itching, vaginal burning, dyspareunia, and edema, and may form a thick or sticky discharge (Sheppard, 2020). It is quite alarming that no new class of antifungals has reached the recipients since the 2000s to treat VVC. The loss of productivity is mainly due to the eukaryotic nature of fungal cells, difficulties with compound permeability across the fungal cell wall and membrane, and a lack of interest from the pharmaceutical industry; the development of novel antifungal agents has generally been slow (Roemer and Krysan, 2014).

Infection and therapy must go hand in hand for a better cure. Once it fails, the global economic burden also increases with the emergence of antifungal resistance. Recent estimates show that the global VVC treatment market value will reach USD 986.5 million. In the next 10 years, the compound annual growth rate (CAGR) will be 4.1% according to a VVC treatment market report (Denning et al., 2018). In parallel, it would be a tremendous challenge for both high-/low-income countries, and there is a pressing need to address this global health-associated risk. Finding solutions through innovations relies on molecular information on the various drivers (predisposition factors), molecular pathways, and associated virulence that affect the pathogen colonization and cause infection in the host. Hence, in this review, we focus on the predisposition factors leading to the virulence of candidiasis, existing treatment strategies with their disadvantages, and how this can be tackled by deciphering possible drug targets and probable mode of drug delivery to cross the preclinical phase by appropriate *in vivo* models.

## 2 Molecular drivers of VVC

VVC recurrence relies on various intrinsic and extrinsic factors. The key host-specific factors, such as the pH of the vaginal environment, age and hormonal status, local defense mechanisms, pregnancy, allergies, psychosocial stress, metabolic issues, immunosuppression, and individual genetic susceptibility, are essential. Additionally, behavioral risk factors including oral contraceptives, overuse of antimicrobials, glucocorticoids, inhibitors of the sodium-glucose co-transporter-2 (SGLT2), intrauterine devices (IUDs), spermicides, and condoms, as well as sexual, hygienic, and dressing habits, demand intervention (**Table 1**) (Patel et al., 2004).

**Table 1:** *C. albicans* virulence pathways controlled by various molecular (predisposition) drivers.

Sl. no.	Proteins encoded	Predisposition factors											References
		pH			Temperature		Oxidative stress	Hormone	Nutrient limitation	Quorum sensing	Glucose	CO_2_	
		Acidic	Neutral	Alkaline	Low	High				Farnesol			
1	Phr1			+									(Fonzi, 1999; Calderon et al., 2010; Popolo et al., 2017)
2	Phr2	+											(Fonzi, 1999; Calderon et al., 2010; Popolo et al., 2017)
3	Efg1		+										(Nie et al., 2010; Glazier, 2022)
4	Rim101			+									(Obara et al., 2012; Yan et al., 2020)
5	Rim21			+									(Yan et al., 2020)
6	Rim8			+									(Yan et al., 2020)
8	Ssb1p					+	+		+				(Cheng et al., 2013)
9	Cyr1											+	(Zou et al., 2010)
10	Ndt80										+		(Hanumantha Rao et al., 2021)
9	Srr1						+						(Mavrianos et al., 2013)
10	Tup1									+			(Kebaara et al., 2008)
11	Hsp90					+							(O’Meara et al., 2017)
12	Pde2									+			(She et al., 2020)
13	Pdr16									+			(Bencova et al., 2020)
14	Flo8											+	(Li et al., 2019)
	Tsa1p						+						(Komalapriya et al., 2015)
	Cap1p						+						(Chien et al., 2019)
15	Hog1						+						(Chien et al., 2019)
15	Gpd2							+					(Kumwenda et al., 2022)
16	Ume6			+									(O’Connor et al., 2010)

'+' indicates the upregulation of the protein expression.

### 2.1 pH

*C. albicans* makes use of its remarkable capacity to respond to variations in nutrition availability, ion or serum concentrations, osmotic pressure, and ambient pH to thrive and spread within the host. Most strikingly, *C. albicans* could colonize and exhibit a normal growth rate on various host niches over a wide range of ambient pH (Brotman et al., 2014). However, in its transition from a commensal to a pathogenic state, the host environment pH changes to slightly alkaline and, to an extent, neutral, like a blood pH of 7.3. The change in the extracellular pH modulates several *C. albicans* genes, viz., Rim101-dependent or -independent pathways (Davis et al., 2000; Vylkova et al., 2011). Among various genes, *PHR1* and *PHR2* (pH-responsive genes 1 and 2) are the first reported Rim101-dependent pH-regulated genes in *C. albicans*. However, the expression of pH-responsive genes is finely regulated, where a pH ≤ 5.5 is required for *PHR1* expression; in contrast, a pH ≤ 5.5 is required for *PHR2*. The pH-dependent control is essential for *C. albicans* to establish pathogenesis in the host niches. Highly comparable glycoproteins that are believed to be attached to the plasma membrane by a glycosylphosphatidylinositol (GPI) are encoded by *PHR1* and *PHR2*, respectively (Davis et al., 2000). *C. albicans* deficient in expressing *PHR1/PHR2* proteins invariably affects its morphogenesis, and cell wall synthesis is pH dependent (Lesage and Bussey, 2006). In addition, the weak organic (lactic) acid released from the host-epithelial or the co-colonized microbes dissociates directly into microbial cells because of their lipophilic characteristics. On internalization, the negatively charged counter-ion accumulates and leads to a rise in turgor pressure, oxidative stress, and the loss of vital cellular elements such as ribosomal RNA and cofactors, an effective antimicrobial against *C. albicans* (Zeise et al., 2021). The vaginal microbiota processes the glycogen released from the vaginal epithelial cells, especially by female hormones, to produce lactic acid. However, during the dysbiosis of the vaginal microbial consortium, even though glycogen levels are high, the pH remains alkaline, and the environment is prone to infections (d’Enfert et al., 2021).

### 2.2 Hormonal imbalance

Estrogen is a steroid hormone that regulates women’s immune systems and overall health (Brotman et al., 2014). Elevated estrogen levels retrench leukocyte infiltration and epithelial cell-mediated antifungal responses. Among the four isoforms of estrogen [estrone (E1), 17β-estradiol (E2), estriol (E3), and 17α-ethynylestradiol (EE2)], E2 is most potent and more associated with many gynecological disorders (Dubey et al., 2005).

Studies have shown that women of reproductive age are more susceptible to candidiasis. Postmenopausal and prepuberty girls are less prone to infection unless they undergo hormone replacement therapy or recurrent usage of oral contraceptives (Phillips et al., 2023). Furthermore, the effects of estrogen on the immune system and the vaginal microbiome can make it more conducive to the growth of *C. albicans*. *C. albicans* estrogen adaptation has been found to increase fungal virulence by suppressing phagocytosis, facilitating the yeast to circumvent the innate immune responses. The enhanced binding of Factor H on the fungal cell surface in the presence of estrogen is the cause of this emergence. A critical component of the innate immune response, the host’s complement system can recognize and assault fungal cells. The fungal cell surface protein encoded by *GPD2* drives the estrogen-induced expression of Factor H. Factor H is a human complement regulatory protein that aids in inactivating the alternative complement system and, thus, preventing the assault on fungal cells (Kumwenda et al., 2022). However, preclinical studies on animal models have shown the role of exogenous estrogen in the fungal infection to persist in the vaginal area. The hormone estrogen promotes the process of keratinization and cornification of the upper layers of the vaginal epithelium, which provides an environment that is favorable for the growth of *C. albicans* (Dennerstein and Ellis, 2001; Willems et al., 2020). Furthermore, pregnancy, antibiotic misuse, and age might cause an imbalance in estrogen levels, boosting susceptibility to infection (Aguin and Sobel, 2015). In this context, estrogen appears to be crucial for *C. albicans* to cause hormone-associated vaginitis, whereas progesterone does not impact the same (Fidel et al., 2000).

### 2.3 Diabetes mellitus

Women with diabetes are more susceptible to VVC owing to increased serum glucose levels. Several case studies show the relationship between women affected with DM and recurrent VVC (Mohammed et al., 2021). DM can lead to systemic candidiasis through diabetic vasculopathy, hypoperfusion and hyperglycemia, and microvascular disease progression, thus damaging the host defense mechanism, leading to fungal growth and adhesion (Guimarães et al., 2012). The inter-relationship of DM and VVC is due to the expression of glucose-inducible surface proteins that resemble the complement system proteins. An increase in the level of such surface proteins weakens the immune system. Furthermore, the reduced neutrophil migration diminishes their functions, including phagocytosis, chemotaxis, and intercellular killing (Sustr et al., 2020).

Blood glucose levels and evasion of the immune system strengthen the major virulence factors in *C. albicans*, like secreted aspartyl protease (Sap), phospholipases, candidalysin, and biofilm formation. Sap proteins can break down various host-related components and aid in generating nitrogen, which is required for *C. albicans* penetration in the host cells (Czechowicz et al., 2022). Similarly, phospholipases target membrane phospholipids to initiate cell lysis and penetration (Ghannoum, 2000). Candidalysin encoded by the gene *ECE1* promotes the influx of calcium ions and lactate dehydrogenase (LDH), impacting membrane and cell damage destabilization (Ho et al., 2019).

### 2.4 Overuse of antifungals

The repeated use of antifungals, especially azoles, polyenes, and echinocandins, as a first-line treatment strategy for VVC can develop resistomes in *C. albicans*, thereby fostering its adhesion and colonization in the epithelial cells to initiate infection (Bhattacharya et al., 2016; Bhattacharya et al., 2020). Overuse of antifungals can lead to a conformational change in the target, overexpression of efflux pumps, modulation of stress responses, and genomic modifications (Cowen, 2008). Also, antifungal overuse can disrupt the normal microflora of the vagina, leading to an increased recurrence of infection. Lack of proper diagnosis and use of antifungals after antibiotic treatment can also be prominent reasons for generating multidrug-resistant strains (Donders et al., 2011).

### 2.5 Other factors

Various other factors, including variations in vaginal temperature, nutrient stress, and immunocompromised state, can lead to the colonization of *C. albicans* in the host epithelial tissue (Willems et al., 2020). The equilibrium of the vaginal microbiota is critically dependent on vaginal temperature. The environment for the growth of *C. albicans* may be more favorable if the vaginal temperature rises (Antley and Hazen, 1988). Likewise, nutrient stress results from a vital nutrient deficiency that sustains a healthy vaginal environment. The immune system needs enough specific nutrients, such as vitamins, minerals, and antioxidants, to perform at its best and maintain a healthy vaginal microbiome. Some nutrient deficiencies might weaken the immune system and make it more challenging to regulate *C. albicans* overgrowth (Rutherford et al., 2019). Also, HIV/AIDS, a few autoimmune disorders, cancer treatments including chemotherapy or radiation, organ transplantation, and long-term immunosuppressive drug use are among the conditions that can lead to immunocompromise. Such immunocompromised women are more prone to *C. albicans* growth and proliferation, which can result in more frequent and severe vaginal candidiasis episodes (Willems et al., 2020).

## 3 Molecular driver-associated virulence determinants

**Figure 1:**
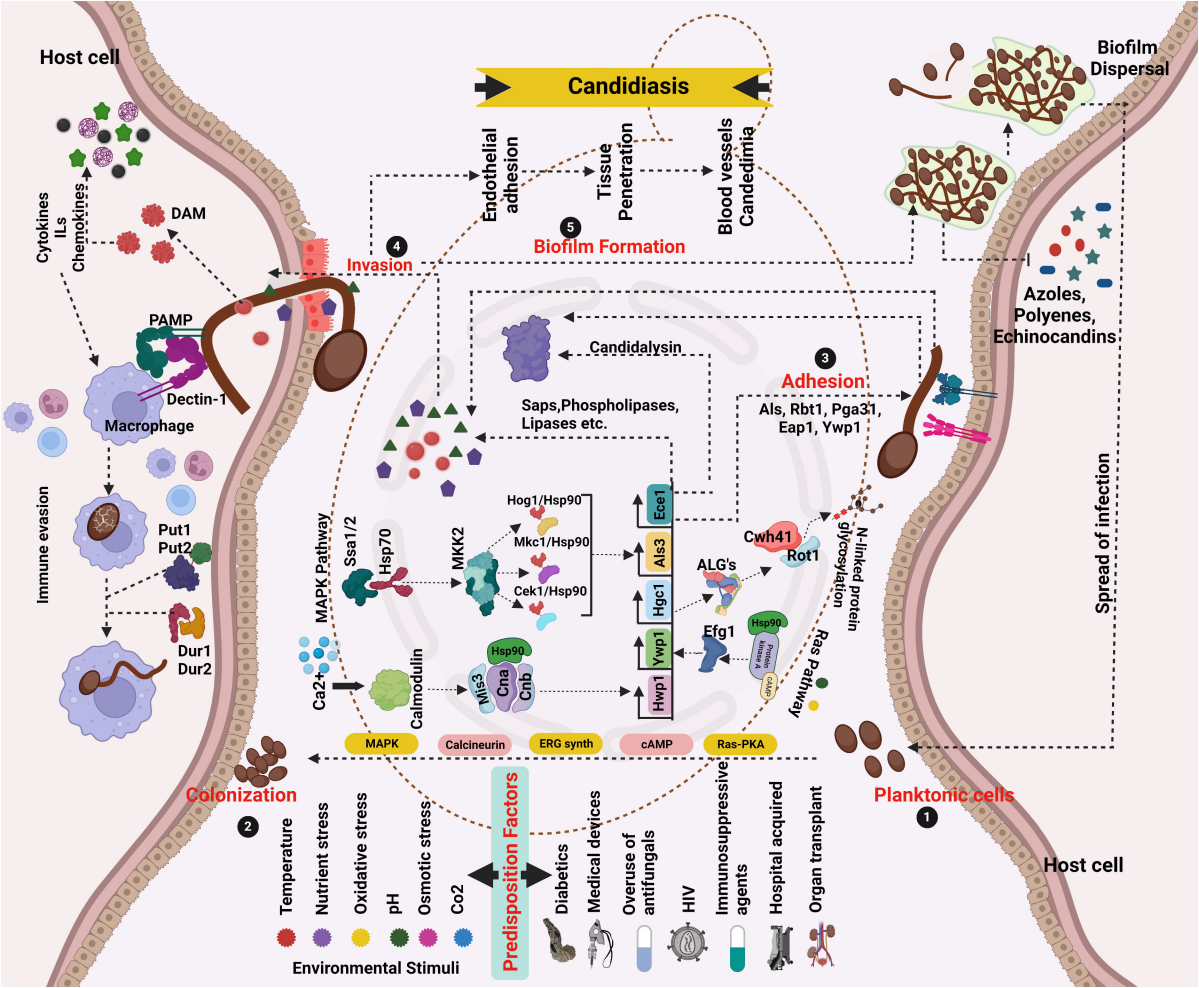
Network pathway analysis of *Candida* pathogenicity and virulence driven by various predisposition factors. (1 and 2) Yeast cells that are planktonic colonize surfaces. Favorable conditions promote overgrowth and adherence (3), where cells stick to host cells using adhesins, and hyphae formation/extension and environmental constraints activate HSPs, signaling, and adaptation pathways that activate genes related to morphology. The pathogenesis of *C. albicans* begins with hyphal development. (4 and 5) Epithelial/endothelial adhesion/invasion with the help of hydrolytic enzymes. Prior to the onset of infection, generate biofilms (6). After the maturation of the biofilm, the dispersed cells from the biofilm localize to other areas leading to the spread of infection. Different kinds of candidiasis are caused by cytolytic proteins and enzymes destroying epithelial and mucosal surfaces (created using BioRender.com).

The dysbiosis of the healthy microbiome in the vagina by various predisposition factors leads to the initiation of the virulence mechanism in *C. albicans.* The evolution of yeast-like fungi to an invasive pathogen depends on many fungal factors along with the host. Such host–pathogen interaction relies on many stages, including adhesion, invasion, biofilm formation, and spread of infection (Willems et al., 2020) (**Figure 1**).

### 3.1 Adhesion

Adhesion is a multifactorial complex process, and with the influence of various predisposition factors, *C. albicans* colonize and adhere to the epithelial cells. The colonization of the pathogen with the host epithelial cells is through non-specific interactions like hydrophobic and electrostatic interactions and effects changes in the central metabolic pathways like MAPKK, Ras-PKA, Calcineurin, and Ergosterol synthetic pathway (Biswas et al., 2007; Moyes et al., 2015).

Following successful colonization, various adhesive proteins will initiate adherence to the receptors on the host. These adhesive proteins are encoded by different families of genes, including *ALS* (Agglutinin Like Sequence)*, HWP1* (Hyphal Wall Protein), and *EPA* (Epithelial Adhesins). *ALS* is the largest among them and encodes eight proteins (*ALS1–7* and *ALS9*) bound to glycosylphosphatidylinositol (GPI). All these proteins mainly have three regions, an N-terminal domain for specific substrates, the core region with tandem repeat sequences, and a C-terminal domain with a GPI anchor sequence (Nobile et al., 2006; Hoyer and Cota, 2016).

The other critical group of adhesins is the *HWP1* protein, which has a characteristic of hyphal formation, with a unique mechanism of action. The N-terminal domain is rich in Gln-Pro, the binding site of host transglutaminase catalyzing the adhesion. A recent study reports that *HWP1* and other adhesins are necessary for rat catheter-associated infections (Maras et al., 2021). Like *HWP1*, *C. albicans* adherence to host epithelial cells and biofilm formation includes Eap1p (Enhanced Adherence to Polystyrene) (Li and Palecek, 2003; Richardson et al., 2018; Samot and Rouabhia, 2021). Also, among the cell wall (GPI-anchored)-associated aspartyl proteinases, Sap10 enhances *C. albicans* adhesion compared to *SAP9*. Studies evidence the opposing effect of both the proteinases in that the deletion of the *SAP9* gene in *C. albicans* increased its adhesion and *vice versa* in the other deleted (*SAP10)* mutants (Albrecht et al., 2006).

### 3.2 Invasion

The invasion of *C. albicans* follows the adhesion promoted by its hyphae, which causes epithelial cell damage, penetration, and host immune system inactivation by adhesion to the host cells. The process of invasions is enhanced by the secreted invasins, especially hydrolytic enzymes, including hemolysins, proteases, lipases, and phospholipases (Mayer et al., 2013). Among them, one crucial family of invasins is *SAP 1–10* encoded by *SAP* genes. *SAPs* are classified into three classes based on their sequence homology by amino acid and specificity for the substrate (Schaller et al., 2003). The *C. albicans SAP* isoenzyme family displays sequence homology that indicates the presence of three distinct clusters: *SAP1* to *SAP3*, exhibiting 67% sequence identity, and a closer association among members *SAP4* to *SAP6*, which share a higher sequence homology of up to 90% (Staib et al., 1999; Naglik et al., 2003). *SAP10* involved in the GPI-linked cell surface anchoring is classified as endopeptidases. SAP is involved in the hyphal formation, penetration, and degradation of defense molecules secreted by the host, like alpha-macroglobulin, collagen, mucin, complement proteins, immunoglobulins, and lactoperoxidase. Among different *SAPs*, SAP1–SAP3 are more crucial for invasion; literature has shown the importance of these proteins, especially SAP3, in the colonization and infection of vaginal epithelial cells (Correia et al., 2010; Ali et al., 2018). Recent research has shown that the gene *ECE1*, which codes for the protein candidalysin, is essential for destroying epithelial cells, the generation of cytokines, and neutrophil recruitment. This hyphae-specific protein has 271 amino acids spread over seven lysine-arginine (KR) repeats. Kex2 protease cleaves the protein into eight peptides. Peptide three (N-SIIGIIMGILGNIPQVIQIIMSIVKAFKGNK) produced can cause lysis and trigger inflammatory reactions, hence named candidalysin (Engku Nasrullah Satiman et al., 2020). During experimental VVC, deletion of *ECE1* or candidalysin reduces immunopathologic indicators of infection (neutrophils, pro-inflammatory cytokines, and alarmins) and tissue damage. Notably, deletion mutants did not exhibit altered colonization or defects in filamentation *in vivo*, proving that candidalysin is necessary for promoting immunopathogenesis during VVC and that hyphae are insufficient to do so (Liu et al., 2021).

Another important class of invasins is phospholipases, a group of hydrolases known for the hydrolysis of cell membrane phospholipids inducing cell lysis and penetration to host tissue. Four classes of phospholipases are known to date, A, B, C, and D, classified based on the ester bond they cleave (Ghannoum, 2000; Bassyouni et al., 2015). Hemolysins and esterase are other virulence-causing enzymes in which hemolysins are necessary for iron acquisition and survival when esterase is known to exhibit cytotoxic effects (Tan et al., 2015).

### 3.3 Biofilm formation

*C. albicans* is well known for its multifaceted biofilm formation process, which begins with the successful adhesion of yeast cells to a surface, followed by developing a discrete colony (Sustr et al., 2020). Next, in the intermediate phase, cells organize and begin producing and secreting extracellular polymeric substances (EPS), which enable the maturation of a three-dimensional structure, forming the biofilm. The mature biofilms of *C. albicans* have a more heterogeneous structure, with blastophores and hyphae enclosed in an ECM (extracellular matrix) made of polysaccharide material. Not only cutaneous or mucosal but also biofilm formation associated with medical devices is also predominated by *C. albicans.* It is also critical to observe that the biofilm cells, after maturation, disperse by forming biofilms in new sites and the host tissue becomes vulnerable to the spread of infections (Cavalheiro and Teixeira, 2018). Several genes, including those involved in protein synthesis, the glycolytic cycle, glycolysis, and response to oxidative stress, are activated in *C. albicans* and persist and spread in the blood candidemia. The yeast spreads from the blood to the other sites of the body, where it causes systemic illnesses. Extracellular hydrolytic enzymes, adhesins, phenotypic flipping, and cytolytic proteins also aid in the spread of candidiasis (Atriwal et al., 2021).

### 3.4 Host immune response

*C. albicans* host interaction and the lifestyle transition from commensalism to pathogenic form activate specific virulence pathways and respond to the change in the host environment. Initially, innate immune cell populations recognize *C. albicans* in different ways. It involves the recognition of conserved pathogen-associated molecular patterns (PAMPs) by several families of PRRs, including the C-type lectin receptors (CLRs), Toll-like receptors (TLRs), RIG-I-like receptors (RLRs), and NOD-like receptors (NLRs) (Shibata et al., 2007). The cell wall of *C. albicans* has two distinct layers: the outer layer, primarily made up of O- and N-linked glycoproteins that contain 80%–90% mannose, and the inner cell wall, which contains the skeletal polysaccharides chitin, β-1,3-glucan, and β-1,6-glucan, which provide cell strength and shape. The β-1,3- and β-1,6-glucans are essential *C. albicans* cell wall elements recognized by CLRs (Munro et al., 1998). Dectin 1 (CLEC7A), a CLR expressed primarily on monocytes and macrophages that induce cytokine production as well as the internalization of the fungus through the establishment of a “phagocytic synapse,” is one of the most extensively investigated β-glucan receptors. Caspase activation and recruitment domain containing-9 (CARD9), spleen tyrosine kinase (SYK), protein kinase C12–16, and the RAF1 kinase signaling pathway are all involved in the process through which Dectin 1 produces intracellular signals (Whitney et al., 2014). The release of neutrophil extracellular traps (NETs) during fungal infection is said to be prevented by signaling via Dectin 1 (Branzk et al., 2014). The relevance of CARD9 is highlighted by the fact that CARD9-deficient mice are more susceptible to invasive candidiasis than wild-type mice and that humans with loss-of-function mutations in CARD9 likewise exhibit greater susceptibility to invasive candidiasis (Glocker et al., 2009). Complement receptor 3 (CR3) is primarily involved in detecting β-glucans by neutrophils, phagocytosis, and destroying unopsonized *C. albicans* (van Bruggen et al., 2009).

Various CLRs, including the mannose receptor, DC-SIGN (CD209), Dectin 2 (C-type lectin domain family 6 member E, CLEC6A), and MINCLE (C-type lectin domain family 4 member E, CLEC4E), are capable of recognizing mannans and mannoproteins (Cambi et al., 2008). *Candida* N-mannan is recognized by the mannose receptor, which is predominantly expressed in macrophages. Thus, signaling pathways are crucial for the generation of pro-inflammatory cytokines, including IL-17 (Netea et al., 2008). *Candida* α-mannan is recognized by Dectin 2, which is mainly expressed on dendritic cells (DCs), macrophages, and neutrophils. Dectin 2 has been linked to the generation of reactive oxygen species (ROS), in addition to its function in regulating T helper 17 (TH17) cell responses (Ifrim et al., 2014). During a *C. albicans* infection, dectin 2 and dectin 3 have been shown to heterodimerize, causing pro-inflammatory responses like the generation of tumor necrosis factor (TNF), IL-1, and IL-6. Both monocytes and neutrophils express the CLR MINCLE, which oversees activating protective responses against *C. albicans*, primarily by starting TNF production (Zhu et al., 2013). While DC-SIGN is found on both DCs and macrophages and recognizes *Candida* N-linked mannan, its activation promotes adaptive immune responses by increasing the expression of cytokines that drive TH cell activation and differentiation (Netea et al., 2008). The receptor, Galectin 3, on the surface of macrophages also detects mannoproteins and triggers the release of TNF, which, in turn, causes mouse macrophages to mount a protective antifungal defense (Linden et al., 2013). Likewise, the immunological response to *C. albicans* involves a complicated interaction between T regulatory cells (Tregs). Studies show that Tregs can stimulate and inhibit immunity while treating *C. albicans* infections. The result of the immune response to *C. albicans* depends critically on the interaction between Tregs and other immune cells, such as Th17 cells. Tregs can control inflammatory reactions and stop tissue damage from an overactive immune system. However, Tregs imbalance can obstruct the removal of *C. Albicans* and impair the protective immune response (Whibley and Gaffen, 2014). Similarly, a class of proteins called pentraxins is involved in inflammatory response by the immune system. C-reactive protein (CRP) and serum amyloid P component (SAP) are well-known pentraxins. The liver produces CRP, an acute-phase reactant, in reaction to inflammation. It attaches to several pathogens, including bacteria, viruses, and fungi, and then activates the complement system to aid in removing these pathogens. The other pentraxin, SAP, recognizes the glucans in the fungal cell wall, leading to opsonization and fungus clearing (Du Clos, 2013).

Although the human immune system is typically quite effective at preventing fungal infections, *C. albicans* adopt various strategies that include PAMPs for evading immune system clearance. The mannan-shielded glucans on the surface of *C. albicans* hyphae are less inflammatory than the yeast forms. Thus, the morphogenetic transition from yeast to a hyphal form of *C. albicans* may alter the immune response against the pathogen during the evasion process. However, the exact molecular mechanisms underlying these processes still need to be explored. *C. albicans* also suppress macrophage nitric oxide generation and phagolysosome maturation (van der Graaf et al., 2005). Furthermore, by causing macrophages to change from the more inflammatory M1 to the less inflammatory M2 phenotype, *C. albicans* can improve its ability to survive. It is interesting to note that *C. albicans* can hijack several PRR pathways. For instance, *Candida*-mediated activation of TLR2 can result in immunomodulatory signals that encourage the development of regulatory T cells and a tolerogenic DC phenotype (Reales-Calderón et al., 2014). Various virulent traits of *C. albicans* in causing the infection can be targeted and can be a better solution for the prevalence of RVVC. However, as a solution, the problem exists in the emergence of multidrug-resistant strains, showing resistance to existing treatment strategies.

## 4 Existing treatment strategies for VVC

Based on the severity of the infection, uncomplicated or complicated VVC, treatment strategies are classified into four different classes of antifungal agents. The various classes of antifungals clinically available include azoles, polyenes, echinocandins, and pyrimidine analogs (de Oliveira Santos et al., 2018) (**Table 2**). Azoles are heterocyclic compounds with ring structures containing at least one nitrogen and target the cytochrome P450 enzyme-lanosterol 14α-demethylase encoded by *ERG11*, producing ergosterol from lanosterol (de Oliveira Santos et al., 2018). Similarly, polyenes are polyunsaturated organic compounds containing at least three single and double carbon–carbon bonds, known to destroy the ergosterol content in the fungal cell membrane, leading to the loss of cell membrane permeability and causing antimycotic activity (Letscher-Bru, 2003).

**Table 2:** Molecular resistance mechanisms to the existing antifungals.

Sl. no.	Antifungal drug classes	Antifungals	Drug target	Gene	Mode of resistance	Reference
1	Azoles	Fluconazole, Clotrimazole, Isavuconazole, Voriconazole, Posaconazole, Miconazole, Ketoconazole, Ecnazole	14α–demethylase (*Erg11*)	*ERG11*	Increased concentration of lanosterol 14α-demethylase—Overexpression of drug targets	(Li et al., 2021)
				*ERG11*	Decreased lanosterol 14α-demethylase binding affinity for the drug—Alteration in drug target	(Li et al., 2021)
				*ERG11, UPC2, TAC1*	Aneuploidy	(Li et al., 2021)
				*ERG11, TAC1, MRR1*	Loss of heterozygosity	(Zare-Bidaki et al., 2022)
				*ERG3*	C5 desaturase inactivation—Affects ergosterol biosynthetic pathway—accumulation of sterols other than ergosterol	(Hosseini Bafghi et al., 2022)
				*CDR1, CDR2, SNQ2, MDR1, TPO3*	Overexpression of efflux pumps	(Rybak et al., 2019)
2	Echinocandins	Caspofungin, Micafungin, Anidulafungin	β1-3 glucan synthase	*FKS1, FKS2*	Alteration of drug targets	(Hosseini Bafghi et al., 2022)
				*ERG2*	Frameshift mutation	(Lee et al., 2021)
3	Polyenes	Amphotericin B,Nystatin	Ergosterol	*ERG3, ERG5, ERG11*	Reduction in ergosterol content	(Hosseini Bafghi et al., 2022)
4	Pyrimidine analogues	5-Flucytosine (5FC)	Nucleic acid biosynthesis	*FUR1*	Alteration in 5-flurocytosine leads to uracyl phosphoribosyl transferase inactivation	(Tortorano et al., 2021)

On the other hand, echinocandins are amphiphilic lipopeptides that inhibit beta-1,3-glucan synthase encoded by *FKS1* and *FKS2* (Letscher-Bru, 2003). Pyrimidine analogues mimic the structure of natural pyrimidines, with a potential antimycotic ability by converting 5-fluorouracil to 5-fluoro deoxyuridine catalyzed by cytosine deaminase and thus interfere with the synthesis of DNA, RNA, and protein (Lee et al., 2021).

Like two sides of a coin, although antifungals are a solution, there also exists the problem of acquired resistance to such antifungals, which is mainly overseen (**Table 2**). The acquired resistance to antifungals refers to the capacity of *C. albicans* to evolve defense mechanisms that lessen their susceptibility to them or make them inactive. The molecular changes often involve mutations ranging from point mutations to chromosomal rearrangements. These mutations can have varied effects on drug resistance, from directly preventing the drug from binding to its target to causing gene expression alterations that encourage physiological conditions that increase drug resistance (Berman and Krysan, 2020). In recent years, there has been an increase in reports of the formation of drug-resistant strains, particularly those that become resistant to several different medications. Additionally, it has been shown that these resistant phenotypes can emerge during an illness and in response to therapy, posing even another risk to patients (Lee et al., 2021; Kalimuthu et al., 2022).

*C. albicans* adapt to various changes in the environment due to their high genetic flexibility. When exposed to antifungals, the yeast cell population undergoes a selection process that favors a group of cells with superior stress tolerance (Lee et al., 2021). The majority of acquired resistance mechanisms fall into two categories: (1) mutations that increase the target’s expression or change its affinity for the drug, and (2) mutations that decrease the amount of drug that accumulates intracellularly by either increasing the biological activity or overexpression of drug efflux pumps (Ksiezopolska and Gabaldón, 2018).

There are several ways *C. albicans* resist azoles, including (1) altering the biosynthesis of sterols to replace ergosterol, (2) overexpressing the target enzyme to increase activity in the presence of the antifungal medication, (3) overexpressing drug efflux pumps to lower the intracellular concentration of the drug, and (4) altering the target gene sequence to decrease the binding affinity. Point mutations in the *ERG11* gene have been linked to the development of resistance in *C. albicans*. Interestingly, 21 of the 140 distinct point mutations for the *ERG11* (lanosterol 14-alpha-demethylase) gene have been directly linked to fluconazole resistance. Also, suppression of the *ERG3* gene expression confers azole resistance (Ksiezopolska and Gabaldón, 2018). Resistance to echinocandins is not as widespread as resistance to azoles, but it is far from uncommon and firmly correlated with prior medication exposure. By mutating specific spots in the *FKS1* gene, *C. albicans* may circumvent the effects of echinocandins. Ergosterol levels in the cell membrane are typically associated with resistance to polyenes. It has been determined that mutations in the genes encoding the ergosterol synthesis-related enzymes *ERG2*, *ERG3*, *ERG5*, *ERG6*, and *ERG11* are the cause of the lower abundance of the enzyme seen in polyene-resistant (Walker et al., 2013). Point mutations in the *FCY1*, *FCY2*, and *FUR1* genes and *C. albicans* deficient to the genes *FPS1* and *FPS2* have all been linked to decreased susceptibility to flucytosine. Variations in *FCY1* and *FUR1* inactivate enzymes involved in the pyrimidine pathway and changes in *FCY2* interfere with drug uptake. The absence of *FPS1* and *FPS2* lowered the drug accumulation in the cell (Chapeland-Leclerc et al., 2005). The emergence of resistance to these existing antifungals shows the need for next-generation antifungals with a novel mechanism of action.

## 5 Possible drug targets: finding a solution to VVC

In accordance with the clinically available treatment regimens, the exploration of medications in clinical trials for vaginal candidiasis encompasses various approaches. The investigation of potential treatments involves the study of *Salvia officinalis*, clotrimazole, and their combination (Ahangari et al., 2019). Similarly, the effectiveness of TOL-463 and ibrexafungerp in treating candidiasis is currently in the preliminary stages of research (Marrazzo et al., 2019; Azie et al., 2020). Additionally, researchers are specifically examining oteseconazole’s potential for managing recurrent VVC (Sobel et al., 2022). Furthermore, the exploration extends to boric acid as a potential therapeutic option for non-albicans yeast infections (Ray et al., 2007). However, with a restricted range of clinically accessible antifungals and ongoing challenges in developing novel antifungal classes, the emergence of antifungal resistance poses a persistent threat to the advancement of antifungal treatments. Multidrug-resistant fungus species have recently emerged, and their incidence has increased, spurring research into new treatments. The clinical limits of off-target effects and drug interactions make it an exciting option to optimize these pharmacological classes to increase fungal-specific, on-target effectiveness (Revie et al., 2018). Given the potential of spreading resistance, the scientific community and the pharmaceutical industry focus on developing antifungals with a novel mechanism of action against a fungal-specific pathway and potentiating the activity of the existing antifungals. Given that up to 80% of hits turn out to be false positives, discovering novel targets specific to fungus has proven difficult (Murphy and Bicanic, 2021).

Exploring possible therapeutic targets (**Table 3**) for candidiasis based on key pathways while considering biological functions and mechanisms is crucial (Spampinato and Leonardi, 2013). It is essential to identify therapeutic targets for candidiasis to treat candidiasis effectively. *C. albicans* central pathways that involve ergosterol biosynthesis, cell wall formation, mitochondrial function, signal transduction pathways, DNA replication and repair, and protein synthesis present promising drug targets for treatment. However, substantial experimental validation of their biological efficacy is required to ensure the effectiveness and safety of these pharmacological targets (**Table 4**) (Mazu et al., 2016).

**Table 3:** Drug targets and virulence-associated molecular pathways in *C. albicans*.

Sl no:	Gene	Protein encoded	Pathways affected																					Virulence	Reference
			Morpho genesis	Ras-cAMP pathway	MAPK Pathway	N-linked manno sylation	O-linked manno sylation	Cell wall remodeling	Host–Pathogen Interaction	Zinc Homeostasis	Calcneurine Pathway	Stress Response pathway	Cell Cycle	Ergosterol biosynthetic pathway	Efflux Pump	Hog MAPKK Pathway	GPI anchoring pathway	Glyoxylate Cycle	Folate biosynthetic pathway	Pyrimidine Biosynthesis	Cytochrome P450 enzyme	Acetate metabolism	MAPK Pathway		
1	*SFL2*	Sfl2		−																				Adhesion, invasion, and cytotoxicity	(McCall et al., 2018)
2	*SFL1*	Sfl1		+																				Adhesion, invasion, and cytotoxicity	(McCall et al., 2018)
3	*EFG1*	Efg1		+																				Morphogenesis, Cell wall gene regulation	(Wu et al., 2020; Araújo et al., 2022)
4	*TEC1*	Tec1			+																			Hyphal gene regulation	(Sahni et al., 2010; Shareck et al., 2011; Mishra et al., 2017)
5	*PIR1*	Pir1					+																	Cell wall integrity	(Martinez, 2004)
6	*HWP1*	Hwp1	+																					Morphogenesis, adhesion, covalent cross-linking between host and *Candida*	(Maras et al., 2021)
78	*CHT2*	Cht2						+																Cell wall integrity	(Zeng et al., 2023)
9	*ALS3*	Als3							+															Adhesion	(Hosseini et al., 2019; Deng et al., 2021)
10	*IHD1*	Ihd1	+																					Hyphal growth, drug tolerance	(Hameed et al., 2020)
11	*UME6*	Ume6	+																					Hyphal elongation and germ tube formation	(Shao et al., 2022)
12	*CSR1*	Csr1								+														Filamentous growth and biofilm formation	(Wang et al., 2022)
13	*RIM101*	Rim101	+																					Morphogenesis	(Nikoomanesh et al., 2019)
14	*CZF1*	Czf1									+													Morphogenesis, cell wall integrity	(Mottola et al., 2021)
15	*CPH2*	Cph2			+																			Colonization, activation of hyphal growth	(Wakade et al., 2023)
16	*FLO8*	Flo8										+												Hyphal growth	(Jin et al., 2023)
17	*NDT80*	Ndt80											+											Multidrug resistance	(Thomas et al., 2022)
18	*ADR1*	Adr1												+										Filamentous growth and biofilm formation	(Cantero and Ernst, 2011)
19	*PMT1*	Pmt1					+																	Cell wall integrity, adhesion	(Dutton et al., 2014; Gómez-Gaviria et al., 2021)
20	*MNT1*	Mnt1					+																	Hyphal growth	(Gómez-Gaviria et al., 2021)
21	*CWH41*	Cwh41				+																		Cell wall integrity, adhesion, hyphal growth	(Gómez-Gaviria et al., 2021)
22	*ROT2*	Rot2				+																		Cell wall integrity, adhesion, hyphal growth	(Dean et al., 2022)
23	*OCH1*	Och1				+																		Cell wall integrity, adhesion, hyphal growth	(Ivanov et al., 2020)
24	*CDR1*	Cdr1													+									Multidrug resistance	(Román et al., 2023)
25	*HOG1*	Hog1														+								Osmotic stress, oxidative stress, and heavy metal stress	(Román et al., 2023)
26	*GWP1*	Gwp1															+							Cell wall integrity; adhesion	(Cavalheiro and Teixeira, 2018)
27	*GWT1*	Gwt1															+							Cell wall integrity; adhesion	(Umemura et al., 2003)
28	*CHS2*	Chs2															+							Filamentation	(Oberdorfer et al., 2012)
29	*ICL1*	Icl1																+						Filamentation	(Cheah et al., 2014)
30	*CYP51*	Cyp51																			+			Cell wall integrity; adhesion	(Moriyama et al., 2014)
31	*PDK1*	Pdk1																					+	Morphogenesis	(Baxter et al., 2011)
32	*CKA1*	Cka1		+																				Regulation of azole susceptibility	(Bruno and Mitchell, 2005)
33	*URA1*	Ura1																		+				Morphogenesis	(Zameitat et al., 2006)

'+' indicates upregulation and '-' indicates downregulation of the protein expression.

**Table 4:** Current inhibitors of the plausible pharmacological targets and biological pathways that are being directly and indirectly targeted. .

Sl no.	Drug targets	Existing inhibitors	Affected pathways													Virulence	Reference
			Mannosylation	ß-glucan synthesis	Hyphal-specific genes	Hog pathway	Ras1-cAMP Pathway	Folate biosynthetic pathway	Ergosterol biosynthetic pathway	GPI-anchoring pathway	Glyoxylate Cycle	Pyrimidine Biosynthesis	Cytochrome P450 Enzymes	Acetate Metabolism	MAPK Pathway		
1	Gwp1	E1210														Adhesion	(Hager et al., 2018; Watanabe et al., 2012)
2	Gwt1	Gepinacin; AX001														Adhesion	(McLellan et al., 2012)
3	Chitin synthase	Nikkomycins														Adhesion	(Oberdorfer et al., 2012)
4	Isocitrate lyase	Mohangamide														Morphogenesis	(Cheah et al., 2014)
5	Cyp51	VT-1598, VT-1129, and VT-1161														Morphogenesis	(Moriyama et al., 2014)
6	Pdk1	KP-372-1														Morphogenesis; adhesion	(Baxter et al., 2011)
7	Sfl2	Cis-2-dodecenoic acid														Adhesion, invasion, and cytotoxicity	(Yang et al., 2020)
8	Sfl1	Cis-2-dodecenoic acid														Adhesion, invasion, and cytotoxicity	(Yang et al., 2020)
9	Efg1	2′-OMethylRNA oligomers; sodium new houttuyfonate (SNH)														Morphogenesis, cell wall gene regulation	(Wu et al., 2020; Araújo et al., 2022)
10	Hwp1	Allicin														Adhesion, host-pathogen interaction	(Khodavandi et al., 2011)
11	Als3	Zinc oxide nanoparticles; N-(oxazolyl methyl)-thiazolidinedione; silibinin														Adhesion	(Hosseini et al., 2019; Marc et al., 2018)
12	Dihydroorotate dehydrogenase	F901318														Morphogenesis	(Oliver et al., 2016)
13	Glucan synthase	Ibrexafungerp														Cel wall integrity, adhesion	(Gamal et al., 2021)
14	Erg1	Eugenol														Filamentation	(Didehdar et al., 2022)
15	DHFR	Benzbromarone														Morphogenesis	(DeJarnette et al., 2020)
16	Ume6	Carbazole derivatives														Hyphal elongation and germ tube formation	(Park et al., 2021)
17	Adr1	14-3-3 (Bmh) Proteins														Filamentous growth and biofilm formation	(Parua et al., 2010)
18	Pmt1	Rhodanine-3-acetic acid														Cell wall integrity, adhesion	(Orchard et al., 2004)
19	Cwh41	Magnoflorine														Cell wall integrity, Adhesion, hyphal growth	(Kim et al., 2018)
20	Cdr1	Apigenin; Apigetrin														Multidrug resistance	(Ivanov et al., 2020)
21	Hog1	Histatin 5														Filamentation	(Herrero-de-Dios et al., 2020)

▬ Repressed ▬ Enhanced ▬ No Influence.

In the era of “antimicrobial resistance”, addressing candidiasis might entail a tactic of utilizing a combined treatment strategy involving specific anti-virulence/anti-infective agents alongside less effective antifungal drugs (Mota Fernandes et al., 2021). More specifically, the anti-infective/anti-virulence agents inhibit virulence traits without affecting the pathogen growth and, thus, do not exert selective pressure on the pathogen to develop resistance. Such anti-infective/anti-virulence drugs may potentiate the existing failed antifungals exhibiting a synergistic effect in fighting infections, an exciting backbone to develop next-generation antifungals. There are several strategies involved in the design and development of next-generation antifungal/anti-infective agents. Antifungal therapeutics from natural sources have drawn attention because they exhibit structural diversity and uniqueness in functional modes of action, making them desirable candidates to thwart the development of drug resistance (Seleem et al., 2017). Several such natural compounds, for example, piperine, cinnamaldehyde, berberine, and curcumin, are known to downregulate the virulence traits of *C. albicans* (Priya and Pandian, 2020; Yong et al., 2020; Rajasekar et al., 2021; Deng et al., 2022). Similarly, quorum-sensing molecules established to target biofilm formation and restore the pathogen’s susceptibility are also gaining attention. The sesquiterpene alcohol farnesol (C_15_H_26_O) was initially identified as a quorum-sensing molecule generated by *C. albicans*. Farnesol is produced by enzymatically phosphorylating farnesyl pyrophosphate, which inhibits the generation of hyphae in a concentration-dependent manner. The inhibition is achieved by negatively regulating the RAS1-cAMP-PKA pathway by targeting *CYR1*. Farnesol has recently become a prospective drug due to its antifungal efficacy (Nikoomanesh et al., 2023). Probiotic vaginal colonization, particularly with *Lactobacillus* sp., has also been widely studied and shown to have a significantly lower risk of VVC and to even treat VVC. Probiotics function by aiding in the vaginal microbiome’s restoration of balance and safeguarding against the overgrowth of *C*. *albicans.* They accomplish this by creating metabolic by-products that stop the growth of infections, such as organic acids, hydrogen peroxide, bacteriocins, and biosurfactants (Fuochi et al., 2019). In addition to probiotics, targeted antifungal therapy may be used as part of personalized medicine depending on the specific *Candida* strain infecting the patient and how they react to various antifungal medications. It deals with the problem of antimicrobial resistance and offers patients therapy options because of resistance to previous antifungal medications (Wu et al., 2022).

Another evolved strategy is the drug repurposing approach that is gaining importance in diseases like cancer and requires expansion in infectious diseases as well. It involves identifying new therapeutic applications for already approved, withdrawn, abandoned, and experimental drugs. It also has the added benefit of reducing the typical drug development period by up to 5–7 years (Ashburn and Thor, 2004). Notably, *C. albicans* are resistant to various classes of antifungal drugs available on the market, including the most recent ones (Pappas et al., 2016). Hence, combining the existing antifungals with newly developed anti-infectives can potentially overcome the problem of drug resistance, by acting through a different mechanism.

In summary, the next-generation antifungals are a novel class of antifungals with new targets with novel therapeutic indications, and old targets with a new therapeutic indication may be the probable first-line approaches (Bouz and Doležal, 2021). Furthermore, the development of a suitable drug delivery system is equally important in the process of drug development, which would increase the biological efficacy of the drugs, or else the nonspecific delivery leads to the accumulation of drugs in the body, causing increased toxicity.

## 6 Vaginal drug delivery systems

**Figure 2:**
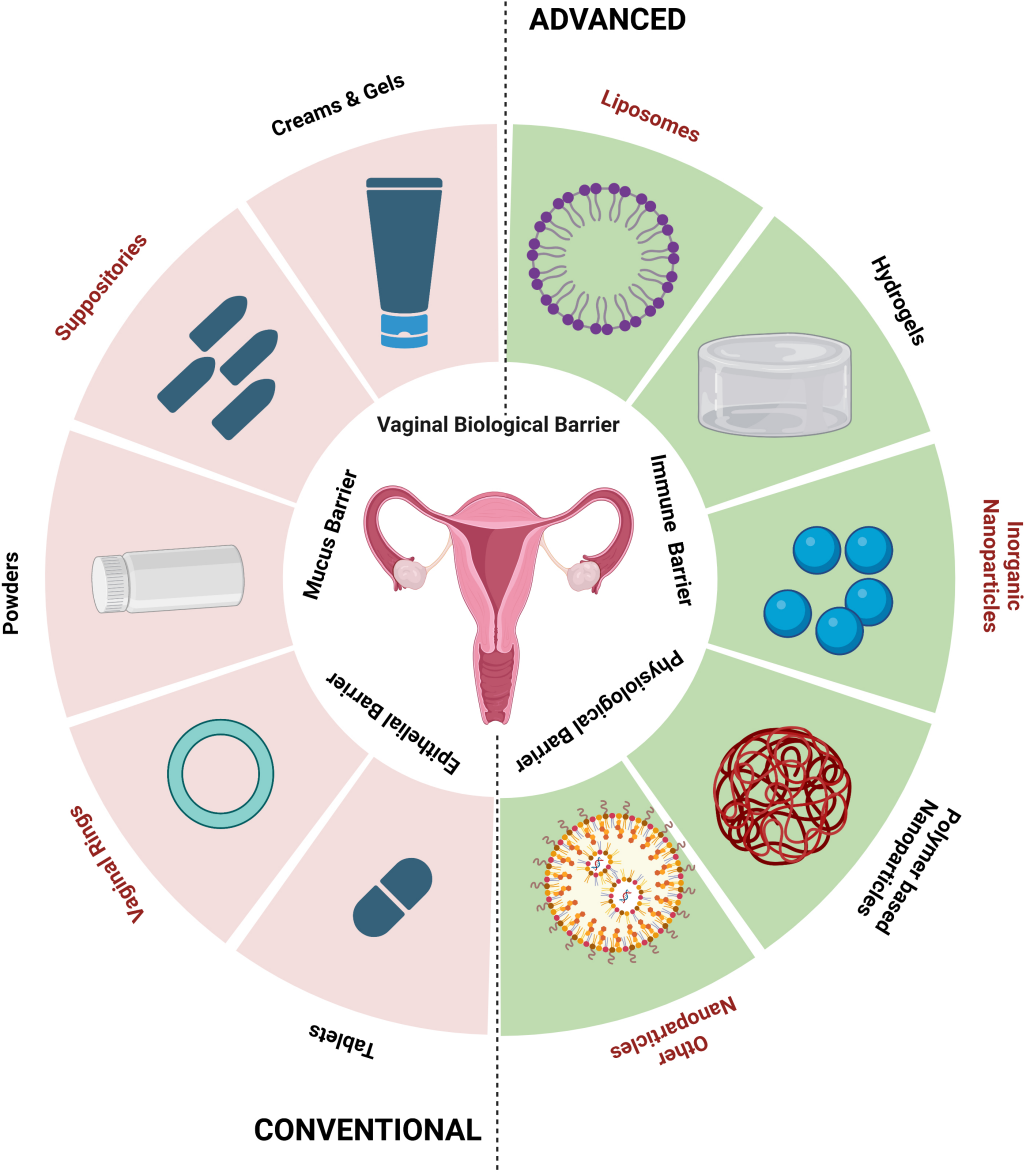
Conventional and advanced vaginal drug delivery systems (created using BioRender.com).

Systems for delivering organ (vagina)-specific medications offer a promising alternative for treating vaginal candidiasis (Pandey et al., 2020). Antifungal drugs, such as topical creams or oral tablets, are used in traditional treatment methods for vaginal candidiasis. These techniques might have limitations, like low patient compliance, systemic adverse effects, and the potential for antifungal resistance (Lírio et al., 2019). Advancements in drug delivery have an array of advantages over traditional approaches and the advancement is the direct delivery of antifungal drugs to the infection site, which enhances local drug concentrations while minimizing systemic exposure (**Figure 2**). Their focused and localized strategy and formulations with sustained release enhance therapy effectiveness, patient comfort, and acceptance (Garg et al., 2020). Drugs administered via vaginal routes are absorbed in three main ways: (1) transcellular, via concentration-dependent gradient; (2) paracellularly, through tight junctions present in between the cells; and (3) vesicular or receptor-mediated transport. The breakdown of drugs in the vaginal lumen and membrane penetration are the two primary phases in drug absorption from the vagina. Therefore, any factor affecting the physiology of the vagina and formulation elements like drug dissolution and membrane transport may change how a drug will be absorbed from vaginal drug delivery devices (Richardson and Illum, 1992). Different physiological factors influencing drug absorption in the vaginal cavity include physiological factors like epithelial thickness of the vagina, vaginal fluid, mucus, pH, and physiochemical factors like lipophilicity, molecular weight, solubility, and degree of ionization (Hussain and Ahsan, 2005).

Using bio-adhesive, nanoparticle-based, and other new delivery systems for vaginal drug delivery is a novel concept **(Table 5**). Bio-adhesive formulations can speed up the healing of fungal infections by administering medications for a longer duration at a steady rate. Using time-release additives can achieve controlled-release medication delivery systems (Osmałek et al., 2021). Other new delivery techniques include phase change polymers, thermoplastic polymers, and mucoadhesive thermo-sensitive gels. Researchers have recently made advancements with hydrogels made with mucoadhesive polymers because of their capacity to interact with the mucus layer and epithelial cells, extending the duration of the drug’s residence time in the vaginal cavity and enhancing drug absorption, thereby achieving good patient compliance (Aka-Any-Grah et al., 2010; Fan et al., 2022).

**Table 5:** Vaginal drug delivery systems.

Sl. no.	Dosage form	Active ingredient	Polymer used	Mode of drug release	Route of administration	Reference
1	Mucoadhesive gel	Calcitermin	Poloxamer 407 and xanthan gum	Sustained	Vaginal	(Bellotti et al., 2023)
2	Bioadhesive hydrogels	Miconazole	Hydroxypropyl cellulose (HPC), Carbopol^®^ 71G-NF or Polycarbophil^®^	Sustained	Vaginal	(Kenechukwu et al., 2022)
3	Nanoformulations	Clotrimazole	N-(2-hydroxy)-propyl-3-trimethylammonium, O-palmitoyl chitosan	Sustained	Vaginal	(FacChinatto et al., 2021)
4	Liposomes	Sertaconazole	Soy phosphatidylcholine, cholesterol, and the cationic surfactant (DDAB)	Sustained	Vaginal	(Abdellatif et al., 2020)
5	Terpesomes	Fenticonazole nitrate	terpenes	Sustained	Vaginal	(Albash et al., 2020)
6	Liposomes	Farnesol & Fluconazole	DPPC and DPPS	Sustained	Vaginal	(Bezerra et al., 2020)
7	Nanoformulation (SLN)	Clotrimazole	Gliceryloleate/Tween 20	Sustained	Vaginal	(Carbone et al., 2020)
8	Microemulsion	AmB and miltefosine	Non-aqueous	Sustained	Vaginal	(de Bastiani et al., 2020)
9	Nanoformulations	Tioconazol and Econazole	Chitosan	Sustained	Vaginal	(Calvo et al., 2019)
10	Bioadhesive tablets	Curcumin	Hydroxypropylmethylcellulose, xanthan gum, and guar gum	Sustained	Vaginal	(Hani et al., 2016)
11	Bioadhesive tablets	Itraconazole	Hydroxypropylmethylcellulose, xanthan gum, and carbopol	Immediate	Vaginal	(Cevher et al., 2014)
12	Bioadhesive tablets	Ketoconazole	Carbomer, hydroxypropylmethylcellulose (HPMC), and hydroxypropyl cellulose (HPC)	Immediate	Vaginal	(Wang and Tang, 2008)
13	Bioadhesive tablets	Ketoconazole	Sodium carboxymethyl cellulose or polyvinylpyrrolidone or hydroxypropylmethylcellulose (HPMC-E50)	Sustained	Vaginal	(Karasulu et al., 2004)
14	Vaginal cream	Terconazole	Butylated hydroxyanisole, cetyl alcohol, isopropyl myristate, polysorbate 60, polysorbate 80, propylene glycol, purified water, and stearyl alcohol		Vaginal	(Sood et al., 2000)

Development of novel drugs with a suitable drug delivery system can only be successful from *in vitro* and, importantly, *in vivo* studies. Most of the drugs and treatment strategies fail in the preclinical validation. Thus, testing and proving these medications in appropriate animal models closely related to the human host can be a better way to reach the beneficiaries.

## 7 *In vivo* models for VVC

The evaluation of the efficacy and safety of a new drug candidate includes *in vitro* and *in vivo* studies that can be carried out throughout all stages of drug development. Studies *in vitro* concentrate on key factors that could affect medication release *in vivo* (Rayner et al., 2021). Basic knowledge of drug pharmacodynamics must be provided, and the selection and application of the right models, as well as accurate data interpretation, are crucial for decision-making and the successful advancement of drug candidates for clinical trials. Understanding a drug’s properties and effects on a living organism requires the use of *in vivo* investigations before the medicine is made available for purchase (Gallo, 2010).

The infectious mammalian models are used to understand the pathogenicity, pharmacokinetics, vaccination attempts involving immunization, and immune responses. Thus, mammalian species would seem most logical to simulate a human host; mice, rats, and rabbits are the earliest recognized animal models (**Table 6**) (Conti et al., 2014). Since the 19th century, these animal models have been explored for *C. albicans-*mediated vaginal candidiasis (Naglik et al., 2008; Conti et al., 2014; Cassone and Sobel, 2016). The mouse model exhibits various benefits, including its inexpensive cost, rapid reproduction, short generation time, and general acceptance in biological and genetic research. Although the mouse’s macroscopic anatomy differs from the humans, both have similar histological characteristics, cyclic estrus/menstrual cycles, and fundamental functions. Also, the murine vaginal microbiota is notably like humans, and although there may be differences between strains, *Staphylococcus, Enterococcus*, and *Lactobacillus* appear to be present consistently (Grasso et al., 1998; Vrbanac et al., 2018). Similarly, like the mouse model, rats have the potential to be an effective vaginal model since they are simple to procure, are inexpensive, and have a reproductive cycle that is similar to that of humans. Wistar and Sprague Dawley female rats are used in many current studies to study the vaginal environment. While the estrus cycle affects the vaginal flora in rats like that in humans, certain bacteria, such as Gram-negative rods, *Streptococci*, and members of the Bacteroidaceae family, may not be present. Generally, a less diversified vaginal microbiome is thought to be more stable and, therefore, healthier in people (Moalli et al., 2005; Levy et al., 2020).

**Table 6:** Standard *in vivo* models for vaginal candidiasis.

Sl. no.	Animal model	Mode of inducing infection	Quantification of infection	Mode of administration of drug	Efficacy of treatment	Reference
1	Female New Zealand rabbits	Intravaginal inoculation	CFU enumeration	Intravaginally	CFU and histopathological studies	(Yang et al., 2023)
2	Female mice (C57BL/6)	Intravaginal inoculation	CFU enumeration	Local	CFU and histopathological studies	(de Araújo et al., 2021)
3	Female BALB/c mice	Intravaginal inoculation	CFU enumeration	Local	CFU and histopathological studies	(de Bastiani et al., 2020)
4	Sprague-Dawley rats	Intravaginal inoculation	CFU enumeration	Intravaginally	Immunological analysis And Histopathology studies	(Abdellatif et al., 2020)
5	Female Wistar rats	Intravaginal inoculation	CFU enumeration	Topical, local, intravaginal	CFU and histopathological studies	(de Oliveira Neto et al., 2021; Permana et al., 2021)
6	*M. mulatta* (rhesus; 4–13 years old) macaques	Intravaginal inoculation	CFU enumeration		Immunological screening, cytokine analysis in vaginal lavage fluid, total antibody analysis in vaginal lavage fluid	(Steele et al., 1999)

For FDA-mandated preclinical assessments of vaginal irritation, the rabbit serves as the gold standard model; as a result, these investigations frequently use rabbit vaginal tissue models. European (*O. cuniculus*) and New Zealand White rabbits are frequent breeds used in these experiments. Compared to some other animal models, the rabbit’s reproductive system and vaginal environment are similar to those of humans, making it a valuable model for researching vaginal candidiasis. Also, the microbial composition of the rabbit’s vagina is like that of humans (Acartürk and Robinson, 1996; Shi et al., 2022). Other large animals like rhesus macaques (*Macaca mulatta*) are also being used for vaginal infection studies. However, these models’ cost, maintenance, and ethical clearance make it difficult (Steele et al., 1999).

## 8 Conclusion and future prospects

Establishing efficient therapies for VVC requires a thorough understanding of the risk factors, pathophysiology, and treatment that can limit the resistance generated. To overcome drug resistance and improve the results for people with VVC, it may be necessary to identify new therapeutic targets and investigate suitable drug delivery modalities. Furthermore, personalized medicine approaches hold promise in the management of VVC. Considering individual genetic susceptibility and host-specific factors, tailored treatment strategies can be developed to improve therapeutic outcomes. The therapeutic measures may involve probiotics, immunomodulatory agents, or combination therapies targeting multiple virulence factors and associated pathways without developing resistance.

## Author contributions

HD: Drafted the article or revised it critically for important intellectual content, which is part of her doctoral studies. AS: Approved the version to be published and agreed to be accountable for all aspects of the work in ensuring that questions related to the accuracy or integrity of any part of the work are appropriately investigated and resolved. All authors contributed to the article and approved the submitted version.
